# What Happened to Patients With Obsessive Compulsive Disorder During the COVID-19 Pandemic? A Multicentre Report From Tertiary Clinics in Northern Italy

**DOI:** 10.3389/fpsyt.2020.00720

**Published:** 2020-07-21

**Authors:** Beatrice Benatti, Umberto Albert, Giuseppe Maina, Andrea Fiorillo, Laura Celebre, Nicolaja Girone, Naomi Fineberg, Stefano Bramante, Sylvia Rigardetto, Bernardo Dell’Osso

**Affiliations:** ^1^Psychiatry 2 Unit, Luigi Sacco University Hospital, University of Milan, Milan, Italy; ^2^Department of Medicine, Surgery and Health Sciences, University of Trieste, Trieste, Italy; ^3^Department of Mental Health, Psychiatric Clinic, ASUGI - Azienda Sanitaria Universitaria Giuliano-Isontina, Trieste, Italy; ^4^San Luigi Gonzaga University Hospital, Orbassano, Italy; ^5^Rita Levi Montalcini Department of Neuroscience, University of Turin, Turin, Italy; ^6^Department of Psychiatry, University of Campania “L. Vanvitelli”, Naples, Italy; ^7^Hertfordshire Partnership University NHS Foundation Trust, University of Hertfordshire, Hatfield, United Kingdom; ^8^Cambridge University School of Clinical Medicine, Cambridge, United Kingdom; ^9^“Aldo Ravelli” Center for Nanotechnology and Neurostimulation, University of Milan, Milan, Italy; ^10^Department of Psychiatry and Behavioral Sciences, Stanford University, Stanford, CA, United States; ^11^“Centro per lo studio dei meccanismi molecolari alla base delle patologie neuro-psico-geriatriche”, University of Milan, Milan, Italy

**Keywords:** obsessive compulsive disorder, COVID-19, Internet-checking, avoidance, suicidal ideation

## Abstract

After the outbreak of Coronavirus disease was declared a pandemic by the World Health Organization, this resulted in extraordinary public health measures to control the infection, such as entire countries being placed under quarantine. The psychopathological consequences of the pandemic and quarantine were anticipated to be of particular relevance, especially in patients with psychiatric disorders such as Obsessive Compulsive Disorder (OCD). Aim of the present report was to describe the impact of COVID-19 pandemics within a sample of Italian patients affected by OCD. Sociodemographic and clinical variables of a sample of 123 OCD outpatients, currently attending three OCD tertiary clinics in Northern Italy, were assessed through telephone and in-person interviews. Patients showing a clinical worsening of OCD represented more than one third of the sample and reported a significant emergence of new obsessions and compulsions phenotypes along with a significant exacerbation of past ones. Moreover, they were more frequently found to experience suicidal ideation, increased Internet checking, sleep disturbances, avoidance behaviors, and work difficulties. A significantly increased need of therapy adjustment and family accommodation was also observed. Further research is warranted to clarify the potential risk and related consequences of the current COVID-19 pandemic on OCD patients.

## Introduction

On 11 March 2020, the COVID-19 worldwide outbreak has been classified as a pandemic by the World Health Organization (1). In order to limit the spread of the SARS-CoV-2 virus, many countries around the world have taken dramatic measures, such as to place entire cities under mass quarantine, with thousands of people living under lockdown (2).

Pandemics are known to have an impact not only on the biological and social context, but also on the psychological one. Thus, the potential usefulness of imposed mass quarantine needs to be watchfully evaluated against the possible psychological consequences (3, 4). A reduction in the availability of routine psychological or psychiatric counselling and timely intervention was observed in some countries. This led, in some places, to the lack of follow-up and medication prescription with potential discontinuation of therapy in psychiatric patients (5, 6).

To date, the recent literature on mental health consequences of COVID-19 has been mainly focused on the effect of social distancing, self-isolation and quarantining on mental health vulnerability (7). Among the several psycho-social consequences of COVID-19 pandemics, the possible worsening of obsessive-compulsive symptoms has been largely overlooked by health service providers, although empirically-based expert guidance for clinicians has recently been published (8). Obsessive compulsive disorder (OCD) is characterized by recurrent and intrusive thoughts or images (i.e., obsessions) associated to behavioural efforts aimed at neutralizing the anxiety caused by obsessions (i.e., compulsions) (9). Given the high impact of OCD on patients’ quality of life and the high rates of psychiatric comorbidities (10, 11), the current COVID-19 outbreak represents a unique challenge both for OCD patients, given the higher disability due to a potential increase in frequency of obsessions and compulsions, and for psychiatrists, since the assessment of “reasonable behaviors” compared to excessive anxiety could be challenging. Since hand washing is considered one of the main precautions against infection, the demand for disinfectants, soaps and gloves has increased, together with insistence on the importance of hygiene, washing and contamination prevention standards. What apparently seem easy rules to follow, may be difficult for patients with OCD, who already have their insecurities about hygienic measures or compulsive cleaning need. Moreover, among the different symptom domains of OCD, obsessions about hygiene and contamination and washing/cleaning compulsions are the most common (12). Besides, although these obsessive and compulsive phenotypes respond better than others to therapy, there could be a higher tendency to recurrence in case of stressful events. In this respect, a recent study by Prestia and colleagues reported that in a sample of OCD patients, the presence of contamination symptoms before the pandemic was associated with a more elevated worsening measured using the Yale-Brown Obsessive Compulsive Scale (13).

The aim of the present report was therefore to assess the impact of COVID pandemic, through a brief cross-sectional interview, on a multicentre sample of OCD outpatients attending three OCD tertiary clinics in Northern Italy, which has been particularly hit by the outbreak.

In this regard, we hypothesize that OCD patients may experience a global OCD worsening (OW), with an increase of pre-existing obsessions or compulsions and the development of new obsessions and compulsions or the switch to different phenotypes. Moreover, we expect that patients with OW may experience a worsening of clinical and behavioural features.

## Methods

Patients affected by OCD of either gender and any age, attending three different OCD tertiary clinics based respectively in Milan, Lombardia region (ASST Fatebenefratelli Sacco, Ospedale Sacco-Polo Universitario), in Torino, Piemonte region (AOU San Luigi Gonzaga) and in Trieste, Friuli Venezia Giulia region (Azienda Sanitaria Universitaria Giuliano-Isontina) were interviewed in-person or by telephone. To limit the transmission of COVID-19 in healthcare settings for patients at higher risk for COVID-19 complications, most of the interviews were conducted by phone, also in consideration of the additional stress related to an in-person interview for OCD patients with contamination fears. In case of patients with specific needs, the interviews were conducted in-person following each hospital’s safety protocols. The three centers previously agreed on each investigated variable and a common database was created. Main questions regarded: gender, age, presence and type of psychiatric comorbidity, obsessions and compulsions main phenotypes, OCD worsening (defined as a clinical worsening assessed during the clinical interview and referred to the last 3 months of pandemic), onset of new obsessions or compulsions, past obsessions or compulsions recurrence, presence of features of inflated responsibility, tic development, pharmacological stability defined as no change in the therapeutic regimens in the three months preceding the pandemics, pharmacological adjustment defined as a change in the therapeutic regimen in the last three months, occurrence of suicidal ideation, increased Internet checking for reassurance, increased family accommodation, increased avoidance behaviors, new sleep disturbances onset, working status and presence of job difficulties. Patients involved in the study had previously provided a written informed consent for research purposes.

In order to compare OCD patients with or without worsening of their OCD and to identify the main features in terms of symptoms and quality of life associated to the clinical worsening, an exploratory analysis was performed, Pearson Chi-squared and ANOVA tests were used, as appropriate, using SPSS 24 for Windows software. The level of statistical significance was set at 0.05.

## Results

The sample included 123 OCD outpatients, distributed as follows: 50 patients from ASST Fatebenefratelli Sacco, Milano (40.7%), 54 patients from AOU San Luigi Gonzaga, Torino (43.9%), 19 patients from Azienda Sanitaria Universitaria Giuliano-Isontina, Trieste (15.4%). Main socio-demographic and clinical variables of the samples are listed in **Tables 1** and **2**. No significant differences in terms of age and gender distribution were found between the three centers, therefore they could be considered comparable.

**Table 1:** Socio-demographic and clinical variables of OCD patients across centers.

	Ospedale Sacco-Polo Universitario, Milano (Lombardia)	AOU San Luigi Gonzaga, Torino (Piemonte)	ASU GITrieste(Friuli Venezia Giulia)	Total sample
N (%)	50 (40.7%)	54 (43.9%)	19 (15.4%)	123
Gender (M:F)	27 (54%); 23 (46%)	31 (57.4%); 23 (42.6%)	10 (52.6%); 9 (47.4%)	68 (55.3%); 55 (44.7%)
Mean Age (years)	41.04 ± 14.34	39.89 ± 13.16	36.79 ± 12.99	39.88 ± 13.59
OCD worsening	22 (44%)	15 (27.8%)	7(36.8%)	44 (35.8%)

Values for categorical and continuous variables are expressed in percentages and mean ± SD, respectively.

**Table 2:** Comparison of socio-demographic and clinical variables of OCD patients with vs without clinical worsening.

	Patients with OW	Patients without OW
N (%)	44(35.8%)	79(64.2%)
Gender (M:F)	24(54.5%):20(45.5%)	44(55.7%);35(44.3%)
Mean age (years)	39.75 ± 13.52	39.95 ± 13.72
Pharmacological Stability	31(70.5%)	60(75.9%)
Pharmacological Changes	31(70.5%)**	**11(13.9%)**
Psychiatric Comorbidity	29(65.9%)	44(55.7%)
New obsessions development	13(29.5%)**	**1(1.3%)**
Past obsessions occurrence	18(40.9%)**	**0(0%)**
New Compulsions development	13(29.5%)**	**0(0%)**
Past Compulsions occurrence	13(29.5%)**	**0(0%)**
Suicidal ideation	4(9.1%)*	**0(0%)**
Internet Checking	23(52.3%)*	**22(27.8%)**
Family Accomodation increase	30(68.2%)**	**11(13.9%)**
Avoidance behaviors increase	29(65.9%)**	**16(20.3%)**
Sleep disturbances	23(52.3%)**	**8(10.1%)**
Working	17(38.6%)	22(27.8%)
Job difficulties	16(36.4%)*	**12(15.2%)**

Values for categorical and continuous variables are expressed in percentages and mean ± SD, respectively. Boldface indicates significant differences between subgroups; **p < 0.005 *p < 0.05. OW, OCD Worsening.

Six percent of the whole sample received an in-person interview, while 94% received a telephone interview. No significant differences emerged between the two subgroups in term of socio-demographic and clinical features.

**Figure 1:**
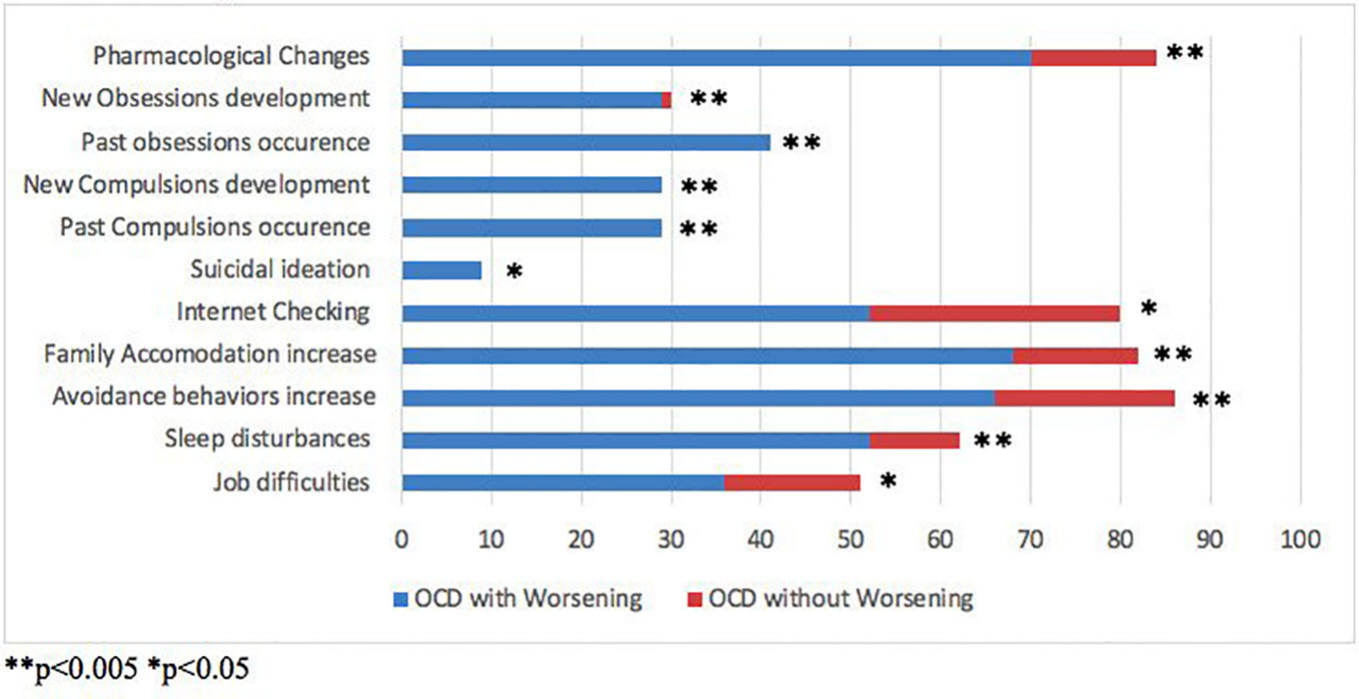
Comparison of socio-demographical and clinical variables between Obsessive Compulsive Disorder (OCD) patients with vs without clinical worsening.

Overall, more than one third of the whole sample reported a clinical OW. The whole sample was then divided in two subgroups: patients with vs without a clinical OW (35.8% vs 64.2% of the total sample). The two subgroups showed comparable mean ages and gender frequencies. No significant differences between patients with and without OW in terms of obsessive phenotypes emerged, the most frequent being violence/harm and multiple phenotypes in both subgroups. Nevertheless, the development of new obsessions (29.5% vs 1.3%; p<0.005) and the recurrence of past obsessions (40.9% vs 0%, p<0.005) was significantly higher in the OW group compared to patients without OW (**Figure 1**).

The most frequent phenotypes of compulsions were washing and cleaning and multiple phenotypes in both subgroups, but no significant differences emerged. However, patients with OW showed a significant increase in both new (29.5% vs 0%; p< 0.001) and past compulsions (29.5% vs 0%; p<0.001) compared to patients without OW. Moreover, worsened patients experienced an increase in avoidance behaviors (OW vs without OW: 65.9% vs 20.3%; p<0.005). No significant differences in terms of inflated responsibility emerged.

No differences occurred in terms of psychiatric comorbidities and pharmacological stability rates, equally represented in both groups with and without OW.

Patients with vs without OW showed a globally impaired clinical picture; in particular, they showed significantly higher rates of pharmacological therapy adjustment (70.5% vs 13.9%; p<0.005), suicidal ideation (9.1% vs 0%; p<0.05), Internet checking for reassurance (52.3% vs 27.8%; p<0.05), family accommodation (68.2% vs 13.9%; p<0.005) and sleep disturbances (52.3% vs 10.1%; p<0.001).

As regards job status, 38.6% of OW patients were working at the time of the interview with no significant differences when compared to the group without OW. However, patients with vs without OW reported significantly higher rates of job difficulties (36.4% vs 15.2%; p<0.05).

A further analysis was made subdividing the whole sample in three age subgroups: 16–30 years; 30–65 years; > 65 years. No differences were found in terms of OCD worsening, nor regarding other clinical features. However, patients in the age range 30–65 years showed higher rates of job difficulties compared to the age ranges 16–30 years and > 65 years (89.3% vs 10.7% vs 0%).

## Discussion

The present study was aimed at giving a snapshot of the clinical status of a multicentric sample of OCD patients during the ongoing COVID-19 pandemics. Main results highlighted that more than one third of the whole multicentric sample presented a OW, assessed through a psychiatric interview, with serious clinical consequences. A first relevant clinical feature related to the OW was the onset of new obsessions and compulsions and the re-experiencing of past obsessions and compulsions, which were absent before the beginning of the pandemic. The onset of new and past obsessions and compulsions could be related to the need of major control against potential contamination or the increase of spare time during the lockdown, leading to an increase in repetitive behaviors. Moreover, the OW group showed increased rates of avoidance behaviours, mostly related to the fear of a possible contamination.

The exacerbation of OCD symptomatology has been well-documented during previous outbreaks, such as Severe Acute Respiratory Syndrome (SARS), Middle East Respiratory Syndrome (MERS), and Influenza (14). According to these evidences, recent studies on COVID-19 and OCD already reported the need to carefully monitor potential relapse of OCD symptoms and their proportionality to the current situation, to prevent backsliding (8, 15, 16).

Functional disability and impairment of health-related quality of life were previously linked to a symptoms relapse in OCD patients (17). In addition, the OW and the loneliness and the social isolation related to the lockdown may have influenced the increased rates of suicidal ideation reported in the OW subgroup (14). OCD *per se* has already been associated with increased levels of suicidality compared with the general population (18).

OW patients showed higher rates of Internet checking, mainly for health reassurance or news checking: this could be interpreted as a response to the current global uncertainty and lack of accessibility to non-priority medical services. The impact of media reports on the exacerbation on some OCD features, such as high intolerance of uncertainty along with frequent and excessive online health search have already been described in OCD patients (7, 19, 20).

Higher rates of family accommodation were found in patients with OW. This result may be directly correlated to both an increase in the frequency and in the manifestation of OC phenotypes co-occurring at the same time. Previous literature showed a correlation of family accommodation with OCD severity and higher functional impairment (21).

The OW subgroup showed a significantly higher need for pharmacological adjustments, also in pharmacologically stable patients. It should be noticed that the OW subgroup also reported higher rates of suicidal ideation and sleep quality worsening: these symptoms may have required further changes in the pharmacological treatment. The revision of the medication status was stated as a treatment priority of OCD during the COVID-19 pandemic (8). This important result should be considered since, especially during the lockdown, the accessibility to non-priority medical services has been difficult. Hence, even patients who needed a therapeutic adjustment, may have had difficulty to achieve it. Moreover, clinical symptoms such as insomnia have been described also in healthy subjects being quarantined (22).

Eventually, the clinical worsening of OCD showed consequences also in the working area, since more than 35% of OW patients reported job difficulties during the last three months. A further analysis on different age classes revealed a higher prevalence of job difficulties in the age range 30-65 years, representing the most numerous subgroups. OCD has a profound impact on patients’ quality of life and working struggles have been previously described in affected individuals (17, 23). The potential risk of contamination in the workplace, the increase in the OC frequency and phenotypes and the higher need of reassurance, along with avoidance behaviours in the workplace may represent triggering factors for the perceived job difficulties.

Main limitations of the present study were represented by the lack of specific psychometric assessment and its cross-sectional nature. In fact, the clinical picture of assessed patients may change in the next months of the current pandemic. Moreover, assessed patients were all living in regions not only particularly hit by COVID-19 but also involved earlier in its management, compared to other European countries.

Further research with specific psychometric measures and follow-up assessment of the sample is warranted to clarify the potential risk and clinical consequences of the current COVID-19 pandemic on OCD patients.

## Data Availability Statement

The data that support the findings of this study are available from the corresponding author, upon request. Requests to access the datasets should be directed to beatricebenatti@gmail.com.

## Ethics Statement

The study was conducted in accordance with the declaration of Helsinki. The patients provided their written informed consent to participate in this study and for the use of their anonymised data for research purposes.

## Author Contributions

Conception and design of study: BB, UA, GM, AF, BD’O, and NF. Acquisition of data/data analysis and interpretation: BB, LC, NG, SB, and SR. Revising the manuscript critically for important intellectual content: BB, UA, GM, AF, BD’O, and NF. All authors contributed to the article and approved the submitted version.

## Conflict of Interest

The authors declare that the research was conducted in the absence of any commercial or financial relationships that could be construed as a potential conflict of interest.

The handling Editor declared a past co-authorship with one of the authors AF.
